# Beneficial Effects of a Moderately High-Protein Diet on Telomere Length in Subjects with Overweight or Obesity

**DOI:** 10.3390/nu17020319

**Published:** 2025-01-17

**Authors:** Blanca De la Fuente, Fermín I. Milagro, Marta Cuervo, José A. Martínez, José I. Riezu-Boj, Guillermo Zalba, Amelia Marti Del Moral, Sonia García-Calzón

**Affiliations:** 1 Department of Nutrition, Food Sciences and Physiology, Center for Nutrition and Research, University of Navarra, 31008 Pamplona, Spain; blancadlf10@gmail.com (B.D.l.F.); fmilagro@unav.es (F.I.M.); mcuervo@unav.es (M.C.); jalfmtz@unav.es (J.A.M.); jiriezu@unav.es (J.I.R.-B.); 2 Consorcio CIBER, M.P. Fisiopatología de La Obesidad y Nutrición (CIBERObn), Instituto de Salud Carlos III (ISCIII), 28029 Madrid, Spain; 3 IDISNA Navarra Institute for Health Research (IdiSNA), 31008 Pamplona, Spain; 4 Department of Biochemistry and Genetics, University of Navarra, 31008 Pamplona, Spain; gzalba@unav.es; 5 Epigenetics and Diabetes Unit, Department of Clinical Sciences Malmö, Lund University Diabetes Centre, Skåne University Hospital, 20502 Malmö, Sweden

**Keywords:** telomeres, high-protein diet, low-fat diet, dietary interventions, calorie restriction, macronutrient distribution

## Abstract

Background and aim: Telomere length (TL) is a key biomarker of cellular aging, with shorter telomeres associated with age-related diseases. Lifestyle interventions mitigating telomere shortening are essential for preventing such conditions. This study aimed to examine the effects of two weight loss dietary strategies, based on a moderately high-protein (MHP) diet and a low-fat (LF) diet on TL in individuals with overweight or obesity. Methods and Results: A total of 164 participants, aged 18–65 years from the OBEKIT trial received the MHP (*n* = 83) or the LF diet (*n* = 81) for 4 months and had TL data for analyses. TL was measured at baseline and after 4 months of the intervention using monochrome multiplex quantitative polymerase chain reaction (MMqPCR). Both groups experienced significant improvements in anthropometric and biochemical parameters after the dietary intervention (*p* < 0.001). The MHP group showed an increase in TL (+0.16 ± 0.13) compared to the LF group (−0.05 ± 0.13) in multiple-adjusted models (*p* = 0.016). An interaction was observed between the sex and dietary group, where women in the MHP group had increased TL (+0.23 ± 0.16) after 4 months compared to women in the LF group (−0.13 ± 0.15; *p* = 0.001); no differences between dietary groups were found in men. This increase in TL for women was associated with an increase in protein intake (*p* = 0.006), measured through dietary questionnaires. Conclusion: This study shows that a MHP diet may have a protective effect on TL during weight loss, particularly in women, potentially contributing to healthier aging. These results highlight the importance of considering macronutrient composition in dietary interventions aimed at preserving TL.

## 1. Introduction

Telomeres, the protective caps at the ends of chromosomes, play a crucial role in maintaining genomic stability. These structures prevent the loss of genetic information during cell division and protect chromosomes from fusion or degradation [1,2]. However, with each cell division, telomeres progressively shorten, eventually leading to cellular senescence or apoptosis once they reach a critical short length [3]. This process of telomere shortening is closely associated with aging and the development of various age-related diseases, including cardiovascular disease, diabetes, and certain cancers [4,5]. Consequently, understanding the factors that influence telomere length (TL) has become a focal point of aging research, as it offers insights into potential strategies for promoting healthy longevity and delaying the onset of age-related pathologies [6,7].

Among the various factors that influence TL, dietary patterns have gained significant attention. Diets rich in antioxidants and anti-inflammatory compounds, such as those found in fruits, vegetables, whole grains, and healthy fats, have been linked to longer telomeres [8,9]. These nutrients are thought to protect telomeres from oxidative stress, a key driver of telomere shortening, by neutralizing free radicals and reducing inflammation [10]. On the other hand, diets with highly processed foods, red meats, and sugars have been associated with a shorter TL, potentially accelerating cellular aging through increased oxidative stress and chronic inflammation [4,8]. The impact of diet on TL underscores the broader role of nutrition in aging, suggesting that dietary interventions could be an effective strategy for promoting telomere maintenance and, by extension, healthy aging [11,12].

High-protein diets, typically characterized by a daily protein intake ranging from 25% to 30% of daily calories, have been widely adopted for weight management and metabolic health [13]. These diets are known to enhance satiety, increase thermogenesis, and help preserve lean muscle mass during caloric restriction (CR), making them a popular choice for individuals seeking to lose weight [14,15]. However, the effects of high-protein diets on TL are not yet fully understood, and the evidence reported remains unclear [16,17]. While some studies suggest that higher protein intake, particularly from plant-based sources, may support telomere maintenance [18], this benefit appears more pronounced among individuals with at least one unhealthy lifestyle factor, such as smoking, heavy alcohol intake, overweight or obesity, or physical inactivity. Additionally, there are concerns regarding the long-term impact of high animal protein consumption on cellular aging and overall health [17].

In contrast, low-fat diets, where fat intake is reduced to less than 30% of daily calories, have traditionally been recommended for cardiovascular health and weight control [19]. These diets emphasize the consumption of fruits, vegetables, and whole grains, which are rich in nutrients that are associated with telomere preservation, such as fiber, vitamins, and minerals [20]. Despite their widespread adoption, the effects of low-fat diets on the TL remain unclear. Some studies have shown that low-fat diets may help preserve TL, possibly due to a higher intake of antioxidant-rich foods commonly associated with these diets [8]. Antioxidants may reduce oxidative stress and inflammation, which are key contributors to telomere shortening. However, other studies have found no significant impact on TL, suggesting that the quality of the low-fat diet and other dietary components may play an important role in these outcomes [8]. The variability of findings highlights the complexity of diet–telomere interactions and the need for further research to elucidate the mechanisms underlying these relationships [21].

Given the growing interest in dietary strategies to promote healthy metabolic aging, previous works have evaluated the effects of dietary interventions on gut microbiota composition and epigenetic mechanisms in the OBEKIT population [22,23]. This study extends this research by specifically investigating the effects of two weight loss dietary strategies, a moderately high-protein (MHP) diet and a low-fat (LF) diet, on TL in individuals with overweight and obesity. By analyzing the impact of these two dietary interventions on TL, we seek to provide insights into the role of macronutrient composition on healthy aging, which represents a novel and clinically relevant area of study.

## 2. Methods and Subjects

### 2.1. Study Design and Participants

**Figure 1:**
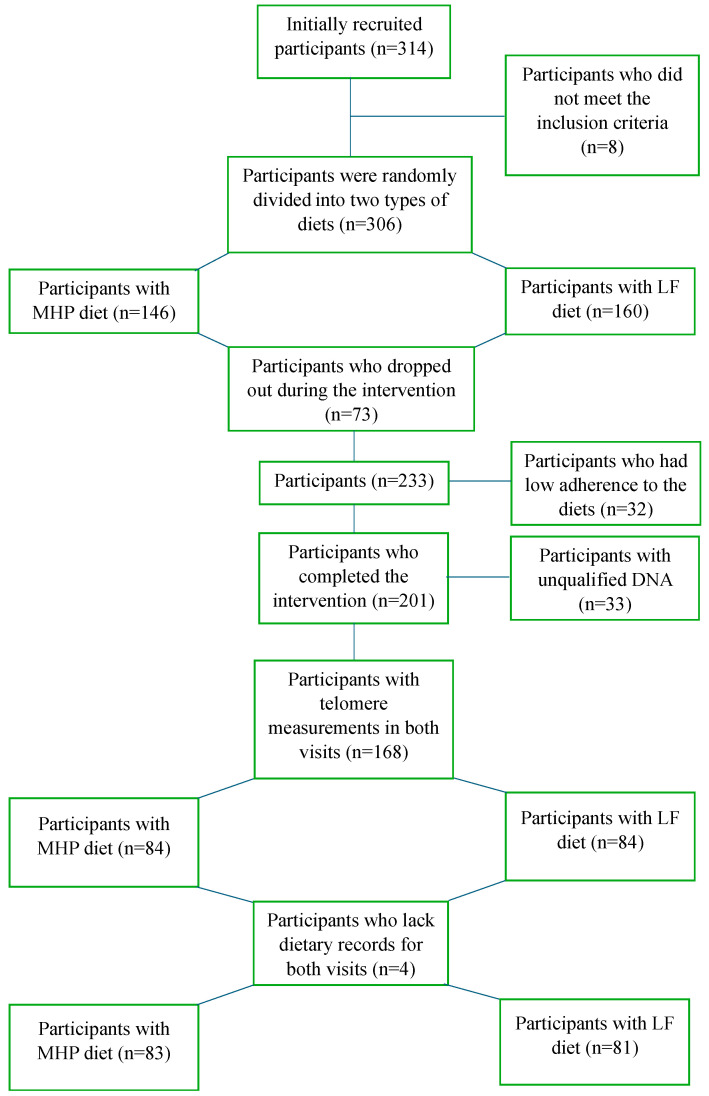
Flowchart of the study participants. MHP, moderately high-protein diet; LF, low-fat diet.

The OBEKIT study, conducted between 2015 and 2017, was a randomized controlled trial (RCT) designed to evaluate the weight loss effects of two dietary interventions—MHP diet and LF diet—on TL in individuals with overweight and obesity [22,23]. Participants were eligible for inclusion if they were aged between 18 and 65 years, had a body mass index (BMI) of 25–35 kg/m^2^, and were free of any chronic diseases or conditions that could influence telomere dynamics or prevent adherence to dietary interventions. Exclusion criteria included pregnancy, lactation, recent weight loss (≥5 kg in the past 3 months), and the use of medications that could affect metabolic or telomere-related outcomes. The flow of participants through the study is summarized in Figure 1. As shown in Figure 1, for the present study, 83 individuals of MHP and 81 of LF with available TL measurements at baseline and after 4 months were included in each of the dietary groups.

All research procedures adhered to the ethical principles outlined in the 2013 Helsinki Declaration [24], in accordance with the European Community’s guidelines for Good Clinical Practice and the legislation and legal Spanish norm that regulate human clinical investigation (RD 561/1993). Approval for the study protocol was granted by the Research Ethics Committee of the University of Navarra (ref. 132/2015) on 29 March 2017. Prior to inclusion in the study, written informed consent was obtained from all participants. OBEKIT is registered as a clinical trial on clinicaltrials.gov under the identifier number NCT02737267.

### 2.2. Dietary Intervention

Participants were randomly assigned to either the MHP or LF dietary group using a specific algorithm designed for the study by MATLAB (http://www.mathworks.com, accessed on 15 January 2025). The MHP diet was designed to provide a macronutrient distribution of 40% of energy from carbohydrates, 30% of energy from proteins, and 30% of energy from lipids; and the LF diet was planned to provide less than 60% of the total energy from carbohydrates, 18% of energy from proteins, and 22% of energy from lipids. Both diets were hypocaloric, with each designed to achieve a 30% energy restriction. Trained nutritionists designed all diets based on a validated food exchange system [25]. Participants were given a menu template with the number of exchanges of each food group for each meal, the list of food exchanges, and structured daily meal plans for 2 weeks. The exchange system offered the participants the most flexibility in diet planning once the concept was learned. In addition, volunteers were instructed to weigh all the food they consumed and all of them received instructions in oral and written format.

During the 4 months of nutritional intervention, nutritionists conducted motivational telephone calls with each participant in order to increase adherence to the dietary advice based on previous trials [26,27]. Moreover, participant’s compliance with the recommended diet was monitored using a 3-day-weighed food record (including 2 weekdays and 1 weekday), which was applied at week 1, week 8, and at the end of the intervention period (week 16). Dietitians provided participants with comprehensive guidance on portion sizes, dietary patterns, eating schedules, and food preparation techniques [28,29,30]. As displayed in Figure 1, participants with low adherence to the diet were excluded from the analyses (*n* = 32).

### 2.3. Phenotypes

Anthropometric measurements such as height (cm), waist and hip circumference (cm), and body weight (kg) were collected in the fasting state by trained nutritionists following validated procedures [31]. The BMI was calculated according to the WHO standards [32]. Body composition was estimated using two methods: bioimpedance (Tanita SC-330, Tanita Corp, Itabashi, Japan) and Dual-Energy X-ray Absorptiometry (DEXA) (Lunar Prodigy, General Electric, Boston, MA, USA). Blood samples were taken after 12 h of fasting to obtain serum and plasma samples. Total cholesterol, high-density lipoprotein cholesterol (HDL-C), triglycerides (TG), serum glucose, and transaminases were assessed using an automatic analyzer (Pentra C200, HORIBA Medical). Low-density lipoprotein cholesterol (LDL-C) levels were estimated using the Friedelwald formula: total cholesterol − HDL-C − (TG/5). Insulin resistance was calculated using the homeostatic model assessment insulin resistance index (HOMA-IR) with the formula: fasting insulin (mU/L) × plasma glucose (mmol/L)/22.5. Serum levels of oxidized LDL (oxLDL) were determined using a solid-phase two-site competitive ELISA (Mercodia AB, Uppsala, Sweden).

### 2.4. Telomere Length Measurement

TL was measured at baseline and after a 4-month intervention period. Genomic DNA was extracted from peripheral blood samples which had been frozen at −80 °C. TL was quantified using a monochrome multiplex quantitative PCR (MMqPCR), as described by Cawthon [33]. The relative TL (T/S ratio) was calculated by comparing the telomere repeat copy number to a single-copy gene; in this case albumin was used. All samples were analyzed in triplicate, as a quality control measure, and any sample showing a variation of more than 10% was reanalyzed. The mean T/S ratio was used for the statistical analyses.

### 2.5. Statistical Analysis

Comparisons were made between the baseline and final characteristics, as well as the differences observed among participants who underwent the intervention, with further stratification by dietary intervention (MHP and LF). The *t*-student dependent test was employed to calculate differences between the baseline and final measurements for each dietary intervention. Furthermore, baseline differences between MHP and LF, as well as the differences observed between MHP and LF post-intervention, were determined using the Mann–Whitney test and multiple regression models. Additionally, differences in TL between both dietary interventions were assessed using ANCOVA, with adjustments for potential confounders such as age, sex, baseline BMI, diabetes status, hypertensive status, dyslipidemia status, smoking status, and total energy intake. The interaction between the dietary group and sex was performed using the likelihood ratio test. The association between changes in TL and changes in protein intake was performed using multiple linear regression analysis. Statistical significance was defined as *p* < 0.05. All analyses were conducted using R studio (R version 4.3.2 for Windows).

## 3. Results

A total of 164 participants completed the dietary intervention and had available TL measurements and dietary records: 83 in the MHP group and 81 in LF group. Table 1 shows the changes in anthropometric, adiposity, and biochemical measurements after the weight loss intervention in each of the dietary groups. All anthropometric parameters improved after the intervention (*p* < 0.001), demonstrating the effectiveness of both diets. Both the MHP and LF groups showed substantial reductions in body weight (−8.31 kg and −9.11 kg, respectively), BMI, waist and hip circumferences, waist-to-height ratio, and body fat percentage (all *p* < 2.2 × 10^−16^). Additionally, the waist-to-hip ratio slightly decreased in both groups (−0.03, *p* < 0.001).

The intervention yielded significant improvements across key biochemical parameters in both the MHP and LF dietary groups. Both groups showed a significant reduction in glucose, cholesterol, LDL-C, TG, and oxidized LDL levels (*p* < 0.001). Insulin sensitivity, as indicated by a reduction in HOMA-IR, also improved significantly (*p* < 0.001). Despite these positive changes, HDL-C levels decreased in both groups (*p* < 0.001). Overall, the MHP and LF diets positively impacted weight loss, body composition, and cardiovascular risk factors. [34,35].

Table 2 displays the dietary characteristics of the participants following a MHP diet or a LF-restricted diet at baseline and after 4 months of the nutritional intervention. Individuals in both groups similarly decreased their carbohydrate intake after the dietary intervention, although the decrease in carbohydrates was greater in the MHP group (−59.61 g/d, *p* < 0.001) compared to the LF group (−33.83 g/d, *p* < 0.001). Protein intake remained relatively stable in the MHP group, whereas the LF group showed a significant decrease (−22.97, *p* < 0.001), especially for animal protein (AP). Fat intake showed significant reductions in both groups (in the MHP group, −33.70 g/d, *p* < 2.2 × 10^−16^, and in the LF group −43.54 g/d, *p* < 2.2 × 10^−16^). Lastly, fiber intake increased only in the LF group (+2.86, *p* = 0.007) with no changes in fiber intake for the MHP group. After four months, changes in all dietary variables were different between both groups after adjusting for the corresponding variable at baseline. Notably, the MHP group’s carbohydrate intake decreased, while maintaining their protein intake and fiber consumption compared to the LF group who had a greater decrease in protein intake and fat intake after 4 months of the intervention.

**Figure 2:**
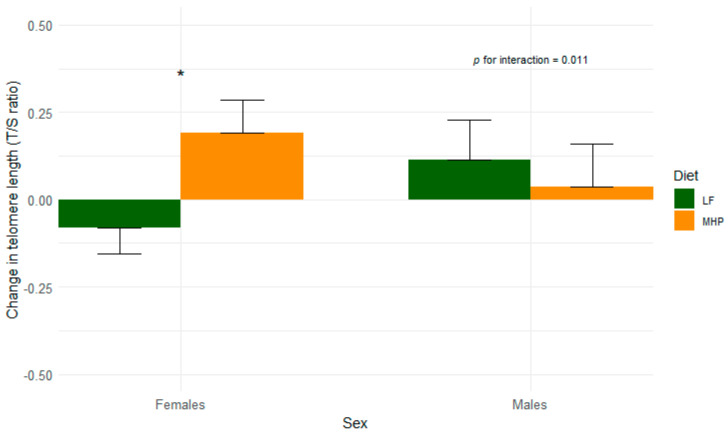
Changes in the telomere length after 4 months of dietary intervention for women and men, separately (*n* = 164). The interaction between the groups of intervention and sex in determining 4 months of changes in telomere length (*p* for interaction = 0.011). MHP, moderately high-protein diet; LF, low-fat diet; T/S, telomere to single-copy gene. Mean and SEM changes in telomere length after 4 months of follow-up in women and men from the intervention group. * *p* = 0.001 between dietary groups for women. All analyses are adjusted for the following confounding factors: age, sex, diabetes status (yes/no), hypertensive status (yes/no), dyslipidemia status (yes/no), smoking status (yes/no), total energy intake (kcal/day), and BMI (in kg/m^2^).

In terms of TL, the MHP group showed an overall increase in TL (+0.16 ± 0.13), compared to the LF group (−0.05 ± 0.13) in multiple-adjusted models (*p* = 0.016) (Supplementary Table S1). Additionally, an interaction was observed between sex and the dietary intervention group in relation to changes in TL (*p* for interaction = 0.011, Figure 2). Female individuals in the MHP group showed a positive change in TL (+0.23 ± 0.16) compared to those in the LF group (−0.13 ± 0.15) in crude and multiple-adjusted models (*p* = 0.024 and *p* = 0.001) (Supplementary Table S1 and Figure 1). No differences in TL were found between both dietary interventions in men, showing that both diets behave the same regarding changes in TL of males.

Table 3 shows the associations between changes in TL and changes in protein intake distributed by sex in crude and different multiple-adjusted models. In female individuals, an association was observed between changes in protein intake and changes in TL in the multiple-adjusted models (β = 0.0087, *p* = 0.006), indicating that an increase in total protein intake was positively associated with a greater increase in TL in females but not in males. Specifically, an increase of 5 g/day in protein intake is associated with a 3.56% increase in TL (95% CI: 2.80% to 4.32%) in women, after adjusting for age, baseline TL, and other covariates. This association was mainly based on AP since a positive relationship between changes in AP intake and TL was observed in females (B = 0.0074, *p* = 0.011). However, for VP, no associations with TL were found in either males or females.

## 4. Discussion

The connection between dietary nutrients, specific foods, and the TL has been extensively studied in nutritional research. However, only a limited number of studies have investigated the link between telomeres with the direct intake of macronutrients in males and females. In this randomized trial, we found that the MHP diet helps to maintain TL compared to the LF diet, specifically in female participants.

Notably, both hypocaloric diets (30% CR) were effective in reducing adiposity and cardiovascular risk factors, with participants achieving a significant 10% weight loss [34,35,36]. These findings align with the results of Sacks et al., who demonstrated that a consistent caloric reduction of 750 kcal per day, regardless of macronutrient composition, resulted in significant improvements in cardiovascular risk factors in adults with overweight or obesity over a two-year period [37]. Similarly, Kraus et al. demonstrated that a 25% CR over two years significantly reduced multiple cardiometabolic risk factors, including BMI, blood pressure, LDL-C, and insulin resistance, in young, non-obese adults [38]. These results were echoed by Most et al., who found that a 25% CR over 24 months in healthy, non-obese adults led to significant improvements in cardiometabolic health [39].

When examining the effect of CR on TL, CR has been shown to have a positive effect on TL, as shown in the EVASYON study. The EVASYON study highlighted the importance of an overall calorie deficit as a key factor in weight loss and improving cardiometabolic health, showing that in adolescents with overweight and obesity, a significant increase in TL was observed after a 2-month energy-restricted diet, particularly in those with shorter baseline telomeres [40]. On the contrary, other studies found no effect of CR on TL, such as the CALERIE™ 2 trial, involving randomized healthy, non-obese men and premenopausal women subjected to 25% CR, which observed no differences in TL over 24 months [41]. Our study, which identified differences in TL outcomes based on macronutrient composition rather than CR alone, suggests that the balance of macronutrients may be crucial in influencing TL. The relationship between dietary macronutrients and TL has been explored in several studies, shedding light on the complexities of how different dietary components may influence cellular aging [3,14,42]. In the literature, a shorter TL has generally been associated with diets characterized by lower protein intake (5%), reduced carbohydrate content (40%), and higher fat levels (55%) [21]. However, it is important to mention that the relationship between individual macronutrients is interconnected, leading to specific macronutrient combinations associated with TL, with these associations varying depending on the overall energy intake. Our study found that an energy-restricted MHP diet led to a significant increase in TL compared to the LF diet, suggesting that a moderately high protein intake may protect against telomere attrition. In contrast, those following the LF diet did not experience changes in TL, despite 30% CR and positive impacts on adiposity and cardiovascular risk factors. In line with this, Koemel et al. reported a positive correlation between moderately high protein intake and TL in US adults [21]. Meanwhile, another recent study among middle-age female nurses found that a higher intake of plant protein was associated with higher odds of healthy aging [43]. Moreover, in a recent study that included 79,294 participants from the UK Biobank, Xu et al. (2024) found that plant protein intake was positively associated with TL, whereas animal protein intake did not show a consistent association with biological aging [44]. Overall, while studies that specifically focus on high-protein diets and TL are limited, the positive effect observed in our study may be due to the potential MHP diet’s ability to reduce oxidative stress and inflammation, and modulate telomerase expression, promoting telomere extension [45].

We found that the MHP dietary group increased their TL after 4 months of follow-up, mainly in women but not in men. Our results are like those from the PREDIMED-NAVARRA trial, which found that a better adherence to a Mediterranean diet was associated with longer telomeres only in women after five years [9]. Moreover, the PREDIMED-PLUS study showed a lower risk of telomere shortening in women following a 3-year lifestyle intervention combining an energy-restricted Mediterranean diet and physical activity [11]. To further explore the impact of high protein intake on TL in women, we specifically analyzed the participants’ protein consumption through dietary questionnaires. We observed a direct association between protein intake itself and TL only in women, confirming that this relationship could be driven by protein consumption. These findings suggest that macronutrient intake, particularly protein, plays a role in telomere dynamics specifically in women. The greater increase in TL in females may also be influenced by estrogen which supports telomere integrity and stimulates telomerase activity, though the relationship is complex and influenced by hormonal fluctuations [46,47]. In this sense, a meta-analysis by Gardner et al. found that women tend to have longer telomeres than men, possibly due to the protective effects of estrogen against oxidative stress [48]. Additionally, other lifestyle factors such as stress, smoking, and physical activity may influence TL differently in men and women, further complicating the interaction between diet, sex, and telomere dynamics. This disparity highlights the importance of considering sex-specific factors in dietary interventions targeting telomere health [48].

One of the main strengths of this study is its novel approach to examining the effects of two dietary weight loss strategies—MHP and LF diets—on TL. Other strengths are the RCT and longitudinal design which provided significant insights and the method of measuring TL (MMqPCR) which enhanced precision by reducing variability. The adjustments for confounding factors further reinforced the robustness of our findings, and the use of accessible blood samples increased the clinical relevance of the results. Nonetheless, some limitations exist: the study’s focus on a specific Spanish population with overweight and obesity may limit its generalizability. Information on sleep patterns, and the family history of obesity of the participants was not considered in this study. Moreover, the 4-month duration may not fully capture the long-term effects on TL, and the limited number of male subjects in the sample may restrict the wider applicability of the findings; however, the significant interaction between the dietary group and sex justified separate analyses to explore biologically relevant differences. In addition to focusing on the effects of dietary interventions on TL, future research should investigate the mechanisms underlying these differences. The interaction between genetic factors and dietary patterns should be explored to determine whether genetic predispositions further influence telomere dynamics. Pro-inflammatory states, pathogen challenges, and epigenetic modifications, which may also impact TL, were not assessed in this study and warrant investigation in future research.

## 5. Conclusions

In conclusion, this study demonstrates that while both the MHP and LF diets are effective in reducing adiposity and improving metabolic health markers, the MHP diet provides a unique advantage in preserving TL, particularly in women. These findings underscore the importance of dietary macronutrient composition in promoting cellular longevity and suggest that higher protein intake may be crucial for slowing the cellular aging process. The results highlight the potential for personalized dietary strategies tailored to individual macronutrient needs, especially for populations at risk of accelerated aging. Further research is needed to explore the long-term impact of these dietary interventions on telomere dynamics and overall health outcomes.

## Figures and Tables

**Table 1:** Baseline vs. final characteristics of the participants following a moderately high-protein (MHP) and a low-fat (LF) hypocaloric diet during 4 months of a nutritional intervention.

	MHP (*n* = 83)				LF (*n* = 81)				*p*-Value ^2^	*p*-Value ^3^	*p*-Value ^4^
	Baseline	Final	Differences	*p*-Value ^1^	Baseline	Final	Differences	*p*-Value ^1^	Basal	Differences	Differences
**Sex, Men%**	26 (31.30)				21 (25.90)				0.554		
**Age**	54.23 (10.18)				54.99 (9.89)				0.620		
**Telomere length, T/S ratio**	1.11 (0.56)	1.25 (0.65)	0.14 (0.68)	0.058	1.07 (0.56)	1.04 (0.51)	−0.03 (0.57)	0.623	0.633	0.132	**0.020**
**Weight, kg**	86.40 (13.27)	78.08 (12.77)	−8.31 (4.02)	**<2.2 × 10^−16^**	87.99 (12.03)	78.88 (11.37)	−9.11 (3.71)	**<2.2 × 10^−16^**	0.289	0.101	0.262
**BMI, kg/m^2^**	31.14 (3.09)	28.11 (3.05)	−3.03 (1.42)	**<2.2 × 10^−16^**	32.28 (3.91)	28.96 (3.77)	−3.32 (1.28)	**<2.2 × 10^−16^**	0.073	0.104	0.410
**Waist circumference, cm**	101.42 (10.45)	92.63 (10.11)	−8.80 (4.51)	**<2.2 × 10^−16^**	102.98 (9.98)	93.36 (10.34)	−9.62 (4.55)	**<2.2 × 10^−16^**	0.194	0.205	0.355
**Hip circumference, cm**	110.72 (7.38)	104.58 (7.78)	−6.14 (3.59)	**<2.2 × 10^−16^**	112.28 (8.21)	105.42 (8.23)	−6.86 (3.59)	**<2.2 × 10^−16^**	0.253	0.123	0.286
**Waist to hip ratio**	0.92 (0.10)	0.89 (0.09)	−0.03 (0.03)	**<0.001**	0.92 (0.09)	0.89 (0.08)	−0.03 (0.04)	**<0.001**	0.915	0.958	0.613
**Waist to height ratio**	0.61 (0.06)	0.56 (0.06)	−0.05 (0.03)	**<2.2 × 10^−16^**	0.63 (0.07)	0.57 (0.07)	−0.06 (0.03)	**<2.2 × 10^−16^**	0.141	0.119	0.250
**Body fat %**	31.88 (6.52)	25.25 (6.65)	−6.63 (3.50)	**<2.2 × 10^−16^**	34.20 (8.85)	26.49 (8.92)	−7.70 (3.42)	**<2.2 × 10^−16^**	0.140	**0.027**	0.110
**Physical activity, METS**	22.78 (20.46)	24.14 (16.85)	1.36 (21.37)	0.563	24.33 (18.61)	21.48 (15.70)	−2.49 (18.76)	0.239	0.464	0.304	0.202
**Glucose, mg/dL**	95.40 (9.89)	91.35 (8.74)	−4.05 (7.98)	**<0.001**	95.30 (11.18)	91.28 (8.72)	−4.01 (7.70)	**<0.001**	0.747	0.817	0.994
**Cholesterol, mg/dL**	216.57 (37.97)	198.47 (38.15)	−18.10 (25.57)	**<0.001**	214.49 (39.92)	192.21 (41.17)	−22.28 (27.83)	**<0.001**	0.731	0.303	0.245
**LDL-C, mg/dL**	142.05 (32.65)	130.82 (33.92)	−11.23 (20.45)	**<0.001**	137.46 (36.29)	123.17 (36.50)	−14.29 (21.58)	**<0.001**	0.361	0.282	0.231
**HDL-C, mg/dL**	53.98 (12.97)	51.01 (11.06)	−2.96 (6.84)	**<0.001**	56.67 (14.06)	51.85 (10.58)	−4.81 (9.30)	**<0.001**	0.189	0.059	0.393
**TG, mg/dL**	102.70 (53.77)	83.17 (47.32)	−19.53 (40.67)	**<0.001**	101.81 (61.66)	85.93 (50.82)	−15.89 (35.71)	**<0.001**	0.453	0.296	0.511
**HOMA-IR**	1.94 (1.45)	1.18 (0.78)	−0.76 (1.42)	**<0.001**	1.91 (1.26)	1.35 (0.85)	−0.57 (1.08)	**<0.001**	0.658	0.555	0.144
**oxLDtg, mg/dL**	45.37 (10.83)	37.29 (11.70)	−8.08 (9.74)	**<0.001**	45.04 (12.90)	36.25 (12.49)	−8.79 (12.25)	**<0.001**	0.526	0.916	0.600

Means (SD) are shown in the table. MHP, moderately high-protein diet; LF, low-fat diet; BMI, body mass index; HDL-C, high-density lipoprotein cholesterol; TG, triglycerides; LDL-C, low-density lipoprotein cholesterol; HOMA-IR, homeostasis model assessment of insulin resistance; oxLDL, oxidized low-density lipoprotein; METS, Metabolic Equivalent of Task; *p*-value ^1^, t-test dependent; *p*-value ^2^, Mann–Whitney test and Chi-squared test comparing baseline values between both diets; *p*-value ^3^, Mann–Whitney test comparing the differences between both diets; *p*-value ^4^, linear regression comparing the differences between both diets adjusted for the baseline respective variable; bold data, statistically significant results.

**Table 2:** Dietary characteristics of the participants with a moderately high-protein (MHP) and a low-fat (LF) restricted diet at baseline and after 4 months of the nutritional intervention.

	MHP (*n* = 83)				LF (*n* = 81)				*p*-Value ^2^	*p*-Value ^3^	*p*-Value ^4^
	Baseline	Final	Differences	*p*-Value ^1^	Baseline	Final	Differences	*p*-Value ^1^	Basal	Differences	Differences
**Carbohydrates, g/d**	201.71 (58.75)	142.10 (33.67)	−59.61 (55.58)	**<0.001**	215.36 (70.81)	181.53 (37.34)	−33.83 (71.41)	**<0.001**	0.257	**0.004**	**<0.001**
**Protein, g/d**	94.27 (21.23)	93.93 (17.87)	−0.35 (18.85)	0.868	92.83 (20.53)	69.86 (12.99)	−22.97 (20.43)	**<0.001**	0.609	**<0.001**	**<2 × 10^−16^**
**AP**	65.03 (18.00)	69.52 (14.92)	4.49 (18.98)	**0.034**	62.67 (19.04)	41.85 (12.55)	−20.82 (20.24)	**<0.001**	0.367	**<0.001**	**<2 × 10^−16^**
**VP**	26.66 (10.47)	24.02 (5.91)	−2.64 (10.13)	**0.020**	27.15 (9.57)	27.66 (6.55)	0.51 (9.83)	0.643	0.515	**0.040**	**<0.001**
**Fat, g/d**	80.90 (26.36)	47.20 (12.15)	−33.70 (24.77)	**<2.2 × 10^−16^**	82.15 (26.89)	38.61 (11.45)	−43.54 (27.82)	**<2.2 × 10^−16^**	0.940	**0.034**	**<0.001**
**MO**	34.89 (12.37)	20.40 (6.00)	−14.49 (11.66)	**<2.2 × 10^−16^**	34.33 (11.52)	16.80 (5.83)	−17.53 (12.33)	**<2.2 × 10^−16^**	0.818	0.163	**<0.001**
**PO**	10.61 (4.57)	9.53 (3.49)	−1.08 (5.64)	0.086	11.10 (5.13)	5.93 (1.74)	−5.17 (5.28)	**<0.001**	0.509	**<0.001**	**<0.001**
**SA**	21.93 (8.46)	9.29 (3.38)	−12.64 (8.67)	**<2.2 × 10^−16^**	23.57 (10.67)	8.39 (3.33)	−15.18 (10.84)	**<2.2 × 10^−16^**	0.454	0.165	0.066
**Fiber, g/d**	21.70 (7.93)	20.68 (5.23)	−1.03 (7.38)	0.208	21.93 (8.61)	24.79 (6.16)	2.86 (9.35)	**0.007**	0.944	**0.004**	**<0.001**

Means (SD) are shown in the table. MHP, moderately high-protein diet; LF, low-fat diet; AP, animal protein; VP, vegetal protein; MO, monounsaturated fatty acids; PO, polyunsaturated fatty acids; SA: saturated fatty acids; *p*-value ^1^, t test dependent; *p*-value ^2^, Mann–Whitney test and Chi-squared test comparing baseline values between both diets; *p*-value ^3^, Mann–Whitney test comparing the differences between both diets; *p*-value ^4^, linear regression analysis to compare the differences between both diets adjusted for baseline respective variable; bold data, statistically significant results.

**Table 3:** Association between changes in TL and changes in protein intake in males and females after the weight loss dietary intervention.

	Crude		Adjusted for Baseline		Sex and Age Adjusted		Multiple-Adjusted	
	β-Coeff (SE)	*p*-Value	β-Coeff (SE)	*p*-Value	β-Coeff (SE)	*p*-Value	β-Coeff (SE)	*p*-Value
**MALES *n* = 47**								
Protein, g/d	−0.0028 (0.0035)	0.420	−0.0032 (0.0039)	0.407	−0.0032 (0.0040)	0.413	−0.0027 (0.0048)	0.575
AP	−0.0020 (0.0033)	0.559	−0.0046 (0.0039)	0.242	−0.0047 (0.0040)	0.247	−0.0011 (0.0037)	0.780
VP	−0.0047 (0.0081)	0.542	0.0049 (0.0108)	0.650	0.0050 (0.0111)	0.656	−0.0058 (0.0103)	0.577
**FEMALES *n* = 117**								
Protein, g/d	0.0006 (0.0029)	0.838	0.0065 (0.0030)	**0.033**	0.0069 (0.0029)	**0.019**	0.0087 (0.0030)	**0.006**
AP	0.0003 (0.0028)	0.906	0.0062 (0.0028)	**0.031**	0.0070 (0.0027)	**0.012**	0.0074 (0.0029)	**0.011**
VP	−0.0003 (0.0067)	0.967	−0.0057 (0.0090)	0.532	−0.0089 (0.0089)	0.320	−0.0013 (0.0070)	0.853

AP, animal protein; VP, vegetal protein; multiple-adjusted: age, sex, diabetes status (yes/no), hypertensive status (yes/no), dyslipidemia status (yes/no), smoking status (never/current), total energy intake (kcal/day), BMI (in kg/m^2^), change in weight, and change in energy intake. Linear regression analysis was performed. Standard error (SE); bold data, statistically significant results.

## Data Availability

Data supporting reported results will be available upon reasonable request and approval by the ethical committee.

## Supplementary Materials

The following supporting information can be downloaded at: https://www.mdpi.com/article/10.3390/nu17020319/s1, Table S1: Changes in telomere length in all individuals and separately in men and women after the weight loss intervention in crude and multiple adjusted models.
