# Supplementary material for: Beneficial Effects of a Moderately High-Protein Diet on Telomere Length in Subjects with Overweight or Obesity
Source: Nutrients. 2025 Jan 17;17(2):319. doi: 10.3390/nu17020319 (PMC11767735; doi:10.3390/nu17020319)
Supplement: Supplementary file 1 [file nutrients-17-00319-s001.zip › nutrients-3392000-supplementary.pdf]

Supplementary Table S1. Changes in telomere length in all individuals and separately in men and women after the weight loss intervention in crude and multiple adjusted models.

**Change in TL**	**MHP (n=83)**	**LF (n=81)**	***p*-value**
	Mean (SEM)	Mean (SEM)	
**OVERALL n=168**			
Adjusted for baseline TL	0.16 (0.06)	-0.04 (0.06)	**0.020**
Further adjusted for age and sex	0.15 (0.06)	-0.05 (0.06)	**0.020**
Multiple-adjusted model	0.16 (0.13)	-0.05 (0.13)	**0.016**
**MEN n= 49**			
Crude model	0.04 (0.11)	0.11 (0.12)	0.658
Adjusted for baseline TL	0.04 (0.11)	0.11 (0.12)	0.648
Multiple-adjusted model	-0.03 (0.20)	0.02 (0.22)	0.784
**WOMEN n=119**			
Crude model	0.19 (0.09)	-0.08 (0.08)	**0.024**
Adjusted for baseline TL	0.21 (0.07)	-0.10 (0.07)	**0.002**
Multiple-adjusted model	0.23(0.16)	-0.13 (0.15)	**0.001**

MHP, moderately high-protein diet; LF, low-fat diet; TL, telomere length. Multiple-adjusted: age, sex, diabetes status (yes/no), hypertensive status (yes/no), dyslipidemia status (yes/no), smoking status (never/current), total energy intake (kcal/day), BMI (in kg/m^2). ANCOVA was performed and adjusted means were obtained.
